# Electroburning of few-layer graphene flakes, epitaxial graphene, and turbostratic graphene discs in air and under vacuum

**DOI:** 10.3762/bjnano.6.72

**Published:** 2015-03-11

**Authors:** Andrea Candini, Nils Richter, Domenica Convertino, Camilla Coletti, Franck Balestro, Wolfgang Wernsdorfer, Mathias Kläui, Marco Affronte

**Affiliations:** 1 Centro S3, Istituto Nanoscienze – CNR, Via Campi 213/a, 41125 Modena, Italy; 2 Johannes Gutenberg Universität-Mainz, Institut für Physik, Staudinger Weg 7, 55128 Mainz, Germany; 3 Graduate School of Excellence Materials Science in Mainz (MAINZ), Staudinger Weg 9, 55128 Mainz, Germany; 4 Center for Nanotechnology Innovation @ NEST, Istituto Italiano di Tecnologia, Piazza San Silvestro 12, 56127 Pisa, Italy; 5 Institut Néel, CNRS and Université Joseph Fourier, B.P. 166, 38042 Grenoble Cedex 09, France; 6 Institut Universitaire de France, 103 Boulevard Saint-Michel, 75005 Paris, France; 7 Dipartimento di scienze Fisiche Informatiche e Matematiche, Università di Modena e Reggio Emilia, Via Campi 213/a, 41125 Modena, Italy

**Keywords:** graphene, graphene based electrodes, molecular electronics, molecular spintronics

## Abstract

Graphene-based electrodes are very promising for molecular electronics and spintronics. Here we report a systematic characterization of the electroburning (EB) process, leading to the formation of nanometer-spaced gaps, on different types of few-layer graphene (namely mechanically exfoliated graphene on SiO_2_, graphene epitaxially grown on the C-face of SiC and turbostratic graphene discs deposited on SiO_2_) under air and vacuum conditions. The EB process is found to depend on both the graphene type and on the ambient conditions. For the mechanically exfoliated graphene, performing EB under vacuum leads to a higher yield of nanometer-gap formation than working in air. Conversely, for graphene on SiC the EB process is not successful under vacuum. Finally, the EB is possible with turbostratic graphene discs only after the creation of a constriction in the sample using lithographic patterning.

## Introduction

The vision of molecular electronics is the exploitation of single molecules as the active units in complex devices offering novel functionalities beyond the present technology [1–3]. To achieve this goal, several technological challenges need to be overcome, in particular how to embed nano-scale objects such as a single molecule in electronic circuits in a reliable way suitable for the mass production of devices [4]. Besides scanning probe techniques [5], to date the most popular approaches are mechanically controllable break junctions [6] and electromigrated junctions [7], with the use of gold as the preferred material for electrodes, due to its noble metal character and (relatively) easy handling. The use of gold, however, brings several well known disadvantages: The mobility of the atoms limits the stability of the junctions and their use for room temperature operations [8–9]. In a three-terminals geometry, the relatively thick electrodes lead to a screening of the gate potential; the atomistic configuration of the metal–molecule–metal junction has a large number of parameters that determine the transport properties which cannot be controlled experimentally, yet [4].

Recently, the use of graphene as electrode material for molecular electronics has been proposed [1,10]. With respect to metallic contacts, graphene offers a planar geometry with a thickness comparable to the molecular size and the strong sp^2^ carbon bonds assure a high mechanical stability even above room temperature. Finally, the possibility to exploit specific functionalizations to attach the molecular units to the graphene electrodes through carbon bonds and/or π-stacking seems to be a promising route to develop well-defined and robust junction configurations. In addition to these advantages, several theoretical papers have investigated the possibility to use graphene as an electrode to contact individual molecules [11–16], predicting interesting specific features such as quantum coherent transport [11], edge effects [13], and suppression of conductance fluctuations [14].

Recent works have successfully made use of graphene for the realization of electrodes in molecular devices [10,17]. Specifically, parallel multi-junctions devices have been fabricated in chemical vapor deposition (CVD) graphene by electron beam lithography and plasma etching [17–19]. In order to address individual molecules the electroburning (EB) technique has been employed on exfoliated few-layer graphene on a substrate, showing electrostatic gating in molecular units at room temperature [10]. More recently, it has been shown how the yield of fabrication of nanometer-sized gaps can be increased from about 50% [20] to more than 95% by performing the EB process under vacuum [21–22]. While this last result is certainly very promising, it has been demonstrated only for single-layer graphene grown by CVD and then transferred on SiO_2_. Therefore it is important to test it also on other types of graphene. In particular, since many envisaged applications require the use of a gate electrode to tune the molecular junctions properties, it seems appealing the use of few-layer graphene, which is still thin but much less gate-dependent than the single layer [10,20].

In this work we compare the EB process in air and in vacuum in few-layer graphene flakes exfoliated on SiO_2_ substrate and we show that the yield of nanometer-gap formation can be increased significantly when working at a reduced pressure. In addition, we report, for the first time, the EB process (in air and under vacuum) also on other types of few-layer graphene, which are better suited for large scale integration, namely multilayer graphene grown on the C-face of SiC [23] and thin discs of turbostratically stacked graphene [24–25].

## Results

### Mechanically exfoliated few-layer graphene

**Figure 1:**
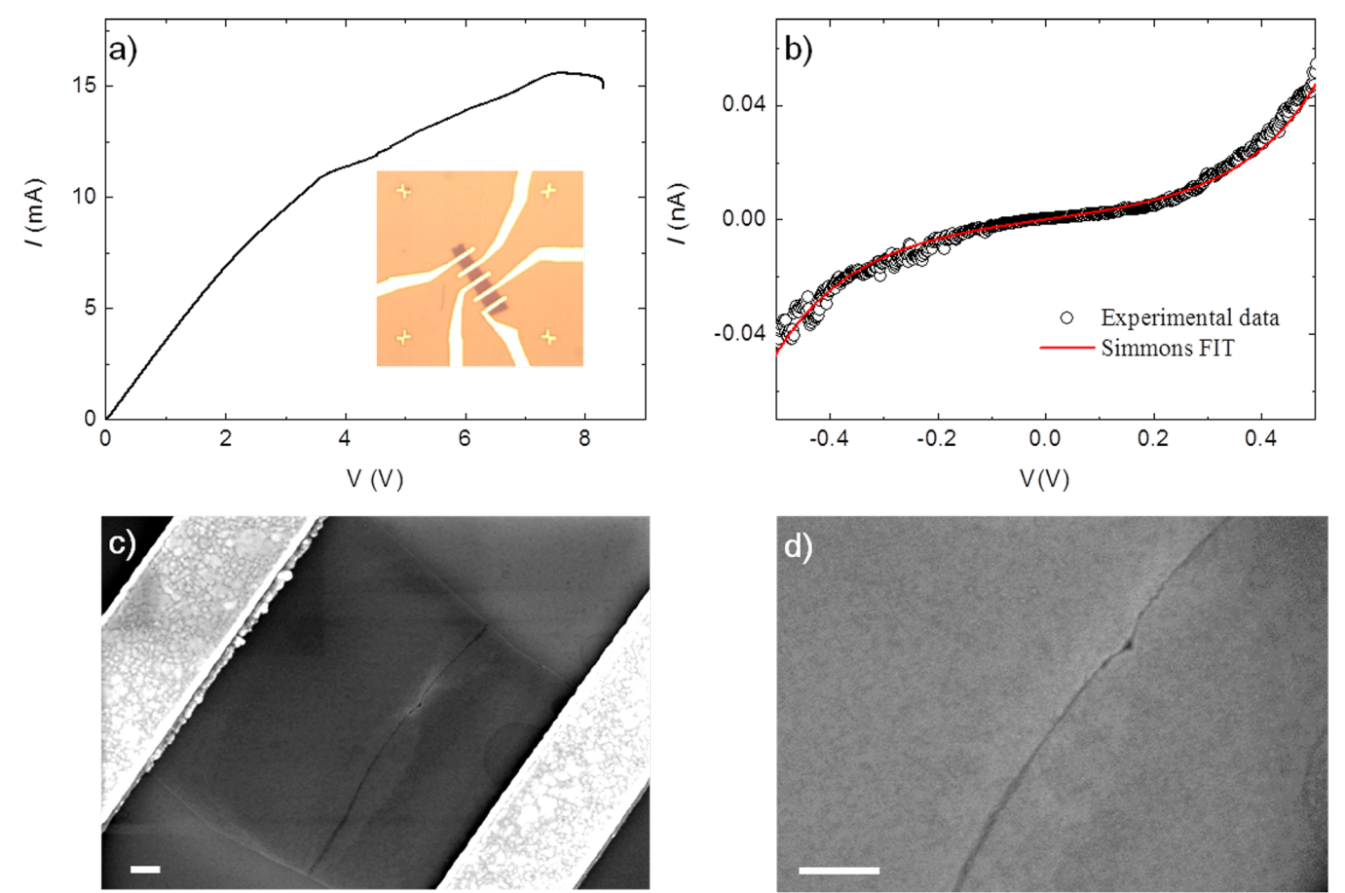
a) *I*–*V* curve recorded for a typical electroburning (EB) process. Inset: optical image of one of the few-layer graphene flakes used (the distance between the crosses at the corners is 45 µm); b) example of an *I*–*V* measurement for a device showing a sizeable tunneling current after the EB process. The black circle are the experimental data and the red line is the fitting according to the Simmons model by using the following parameters: gap size *d =* 1.83 nm; junction area *A* = 5 nm^2^; barrier height Φ = 0.6 eV; c) and d) corresponding SEM images showing the opening of a gap that is a few nanometers wide (scale bars: 300 nm).

We first consider the case of few-layer graphene flakes obtained by the mechanical exfoliation technique (see Experimental for more details). A typical flake is shown in the inset of Figure 1a. Several electrical contacts are fabricated on the same sample, leading to a certain number of nearly identical graphene junctions (same layer thickness, roughly the same geometrical parameters). For each flake we processed approximately half of the junctions under ambient conditions (room temperature and in air) and the other half under vacuum (pressure below 10^−4^ mbar) so that the final comparison between the results in air and under vacuum is made independent on the geometrical factors. In total, we measured 23 graphene flakes of thickness of 1–20 layers, corresponding to 115 junctions.

To open a nanometer-size gap in the graphene devices, we polarized the junctions with a single voltage ramp with a fast feedback loop in order to stop the current immediately after the opening of the junction. We used the same method previously employed for the electromigration of gold nanowires [26]. A typical example of the process is visible in Figure 1a. Above a certain voltage value, the *I*–*V* curves become strongly non-linear, probably related to the removal of contaminants induced by the current annealing [10,20–21,27]. Increasing the voltage further, the true EB process develops. At high temperatures, induced by the Joule heating, the carbon atoms react with oxygen until the device breaks and an abrupt increase of the resistance is observed [10]. When the measured resistance overcomes a fixed value corresponding to the complete formation of an open gap in the device, a feedback control of our electronics restores the voltage to zero very rapidly (<100 µs). The complete process takes approximately 10 to 20 s.

After the EB process, the device is characterized by an *I*–*V* measurement keeping the bias voltage below ±1.5 V to avoid any modification of the gap [10,28]. An example of a device displaying a sizeable tunneling current in this range is given in Figure 1b. Figure 1c and Figure 1d show the corresponding scanning electron microscope (SEM) images for the device after the EB process. The opening of the gap, which can be as small as few nanometers, is visible. The image indicates that the reaction starts at the edges of the central part of the device. Indeed, the gold contacts act as a thermal reservoir dissipating the heat and the central part is therefore the hottest part of the device. In addition, the edges of the flake, which are characterized by nonsaturated carbon bonds, are likely the most reactive point to initiate the process.

**Figure 2:**
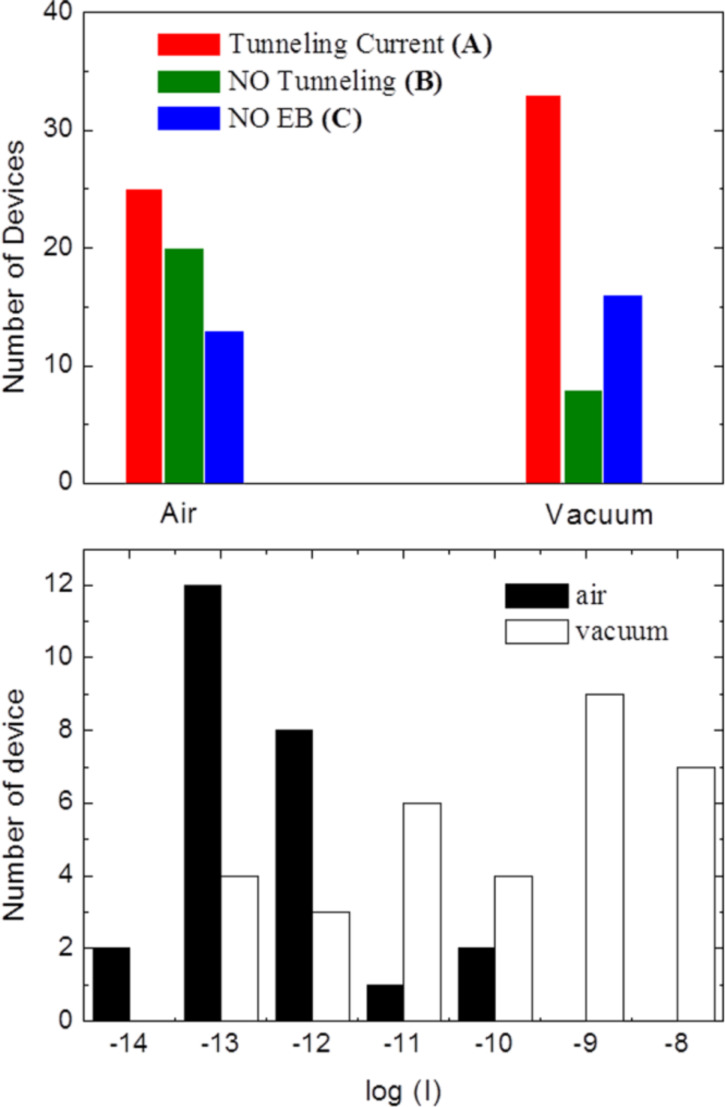
top) Number of measured devices displaying the following behavior: A) sizeable tunneling current (*I* > 10^−13^A @ 1.5 V) after the EB process (red); B) no tunneling current after EB (green); C) no EB for the maximum currents and/or voltages available. Results related to both procedures, in air and under vacuum, are presented. Bottom) Logarithm of the current (in amperes) measured at *V* = 0.5 V for the devices of group A: the measured current is significantly different if the EB process is carried out in air (black histograms) or under vacuum (white histograms).

In order to characterize the gap size in more details, we fit the *I*–*V* curves according to the Simmons model [29] (see Supporting Information File 1 for more details), using the gap width *d*, the junction area *A* and the height of the tunnel barrier Φ as the fitting parameters. For the fit shown in Figure 2b we used *d* = 1.83 nm, *A* = 5 nm^2^, Φ = 0.6 eV. It is known that the value of *d* is a quite robust fitting parameter, while the other two are not, since good fits are still possible even with very different values. Indeed, we can fit our data with almost the same accuracy by using a very broad range of values for *A* and Φ (see the Supporting Information File 1 for some examples) but the optimal value for *d* is always between 1.5 nm and 2.5 nm. For all of our measured devices for which we observed a tunneling current at low bias voltages (also for the ones made of different type of graphene, see following sections) the values for *d* are in the range from 0.5 nm to 3 nm. This narrow distribution, which is expected since in the model the current depends exponentially on the width of the gap, is a strong indication that we can obtain electrodes with distances in the true nanometer range.

In the following, we discuss the yield of this EB process, with particular attention on the differences encountered between air and vacuum working conditions. In total, 58 junctions were processed in air and 57 under vacuum and they have been divided in three categories depending on the *I*–*V* characterization after the EB process. The first class (A) is composed by the devices showing a sizeable tunneling current (*I* > 10^−13^ A) within the range of ±1.5 V. In these devices, the EB process created a gap with sizes of a few nanometers. In the second group (B) we consider junctions that underwent an EB process but no tunneling current is observed in our measurement range. These are “open” devices, with a gap larger than about 10 nm, which can be still useful for contacting long molecules or nanoparticles. Finally, the last group (C) comprises those devices that did not break under the maximum voltage/current we applied. The results of these studies are summarized in the upper panel of Figure 2.

Considering the junctions processed in air, 25 of them (ca. 43%) exhibited a tunneling current after the EB, in 20 (ca. 35%) no detectable current was measured and finally 13 (ca. 22%) did not break during the process. Such a yield is comparable with what has been found by Burzurì et al. [20], but with a different electroburning procedure (i.e., feedback controlled). Moving to the devices electroburned under vacuum, in 33 junctions (ca. 58%) we measured a sizeable tunneling current after the process, while only in eight (ca. 14%) we did not find any measurable current. Finally for 16 (ca. 28%) of them, the EB process was not possible under our experimental conditions. The difference in yield between the process in air and under vacuum is evident.

Interestingly, the tunneling currents measured after the EB process generally display a much higher value when the process is carried out under vacuum than in air, as shown in the bottom panel of Figure 2, in which we plot the current measured at *V* = 0.5 V in different devices. We stress that the *I*–*V* tests after the EB process were repeated also under different conditions (air or vacuum) and we did not find a significant dependence on the measurement conditions (only the conditions of the EB process are relevant). Since the tunneling current is inversely proportional to the size of the gap, we conclude that under vacuum conditions the EB is more controllable, leading to higher yield of success and generally smaller gap sizes.

It is also worthy of note that the number of devices that were not burned is almost the same for air and vacuum conditions. We noted that very thick or very large flakes led to devices displaying very small two-probes resistance (≤200 Ω), which did not undergo the EB process for our maximum applied voltage (10 V) and/or led to the saturation of our current-meter (30 mA). Indeed, the largest part of the devices falling in the category C of Figure 2 is made by junctions belonging to the same thick or large flakes.

If we consider only the flakes that were effectively electroburned, i.e., neglecting devices falling in category C, the difference in yield between the EB process in air and in vacuum is even more evident: the rate of success (junctions displaying tunneling current after EB) is 55% in air and 80% in vacuum. In addition, the gaps obtained by the vacuum process generally display much larger currents implying a smaller size of the gap itself. Similarly, an improved yield in the fabrication of nanometer-sized gaps in graphene junctions by changing the environment conditions from air to vacuum was also reported by Nef et al. [21] who, however, considered only graphene monolayer devices.

**Figure 3:**
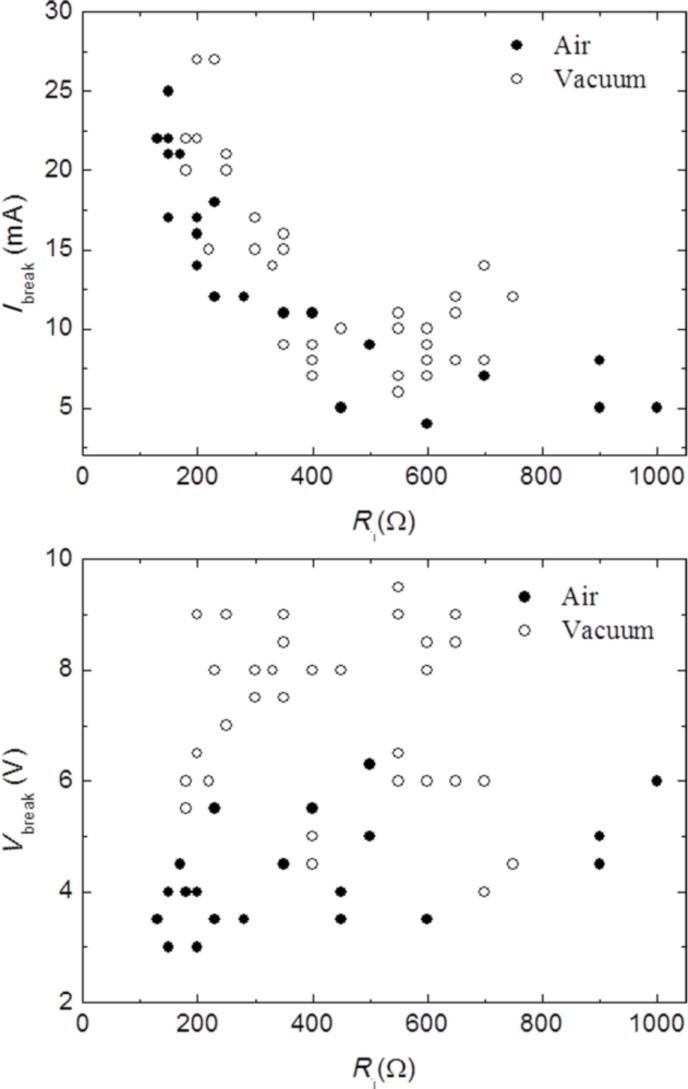
(top) *I*_break_ current at which the EB process was observed in air (filled dots) and under vacuum (open circles) as a function of the initial resistance of the device. (bottom) Corresponding *V*_break_.

In order to analyze further the dependence of the EB process on the environment conditions, in Figure 3 we show the current *I*_break_ (upper panel) and the voltage *V*_break_ (lower panel) at which the EB process occurs as a function of the initial resistance of the junctions for both the working conditions (air and vacuum).

We found that the current is generally similar in the two cases (air or vacuum) and it always scales with the initial resistance. On the other hand, the voltage at which the device rupture is found does not show a clear dependence on *R*_i_ while it is clearly higher when the process is performed under vacuum. The dependence of *I*_break_ on *R*_i_, which is primarily determined by the number of layers of the flakes (inversely proportional), suggests that the EB occurs at a constant current density (which only depends on the geometry of the device), regardless of the environment conditions, and in agreement with previous works [10]. On the other hand, the higher voltage values (at a given current) found when working under vacuum indicate a higher resistance for the devices at the breaking point. This can be related to a different efficiency of the removal of contamination induced by the current annealing in vacuum with respect to air [27]. We will return to this point in the Discussion section.

### Epitaxial graphene on the C-face of SiC

**Figure 4:**
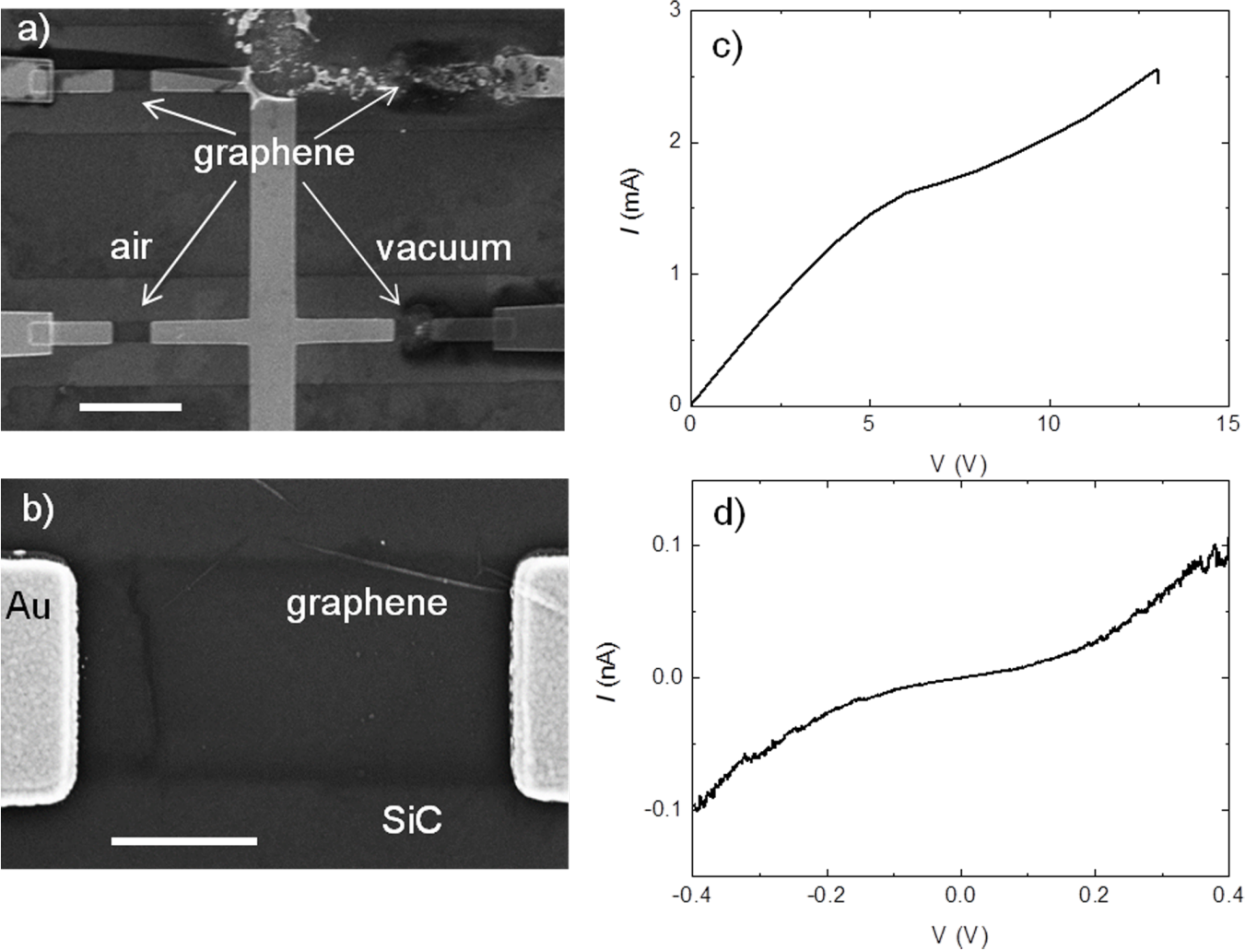
a) SEM image of epitaxial graphene devices after the EB process in air (left) and under vacuum (right). The scale bar is 5 µm. b) Magnification of the open gap in an air-processed device. Scale bar is 1 µm. c) *I*–*V* curve recorded for a typical electroburning (EB) process in air. d) Example of an *I*–*V* measurement after the EB process in air (no current is measured in the *I*–*V* when the EB process is performed under vacuum).

The EB procedure has been repeated also on two-terminal junctions made of graphene epitaxially grown on the C-face of SiC through thermal decomposition in an argon atmosphere [30]. Here the graphene layers are found to grow in a turbostratic fashion, in which each layer is rotated by a certain angle with respect to both adjacent layers. This can be regarded as a large-area few-layer graphene (we found an average of ten layers from Raman measurements, see Experimental section and the Supporting Information File 1 for more details on the sample growth and characterization), which should display a smaller gate dependence with respect to single layer graphene. In total, we processed twelve junctions in air and nine under vacuum. When working under ambient conditions, all the junctions underwent the EB process and an example of the *I*–*V* curve is shown in Figure 4c. In particular, a SEM inspection after the process revealed that the rupture is always in the graphene junction (see the devices on the left in Figure 4a and Figure 4b). After EB, seven junctions displayed a sizeable tunneling current similar to what is shown in Figure 4d. A completely different scenario is found for all the devices processed under vacuum conditions: We did not found any signature of rupture up to very high voltages (*V*_break_ > 40 V for vacuum processed devices, while it is in the range 10–15 V when working in air) and no detectable currents were ever measured after the EB process. Interestingly, the SEM images revealed that the rupture is always at the metal contacts and not in the graphene devices (junctions on the right in Figure 4a).

### Turbostratic graphene discs

We also tested the EB process in multilayered graphene microstructures, employing thin discs of turbostratically stacked graphene (TG, see Experimental for details on the preparation). These discs are comprise up to 100 graphene layers exhibiting a rather large charge carrier mobility in the range of 10^5^ cm^2^/V·s which typically leads to a resistivity of around 3.5 Ω·µm in untreated discs [24]. They can be easily deposited on a substrate (SiO_2_ in our case) in large quantities (hundreds of discs with a diameter of about 1–2 µm in a single deposition).

**Figure 5:**
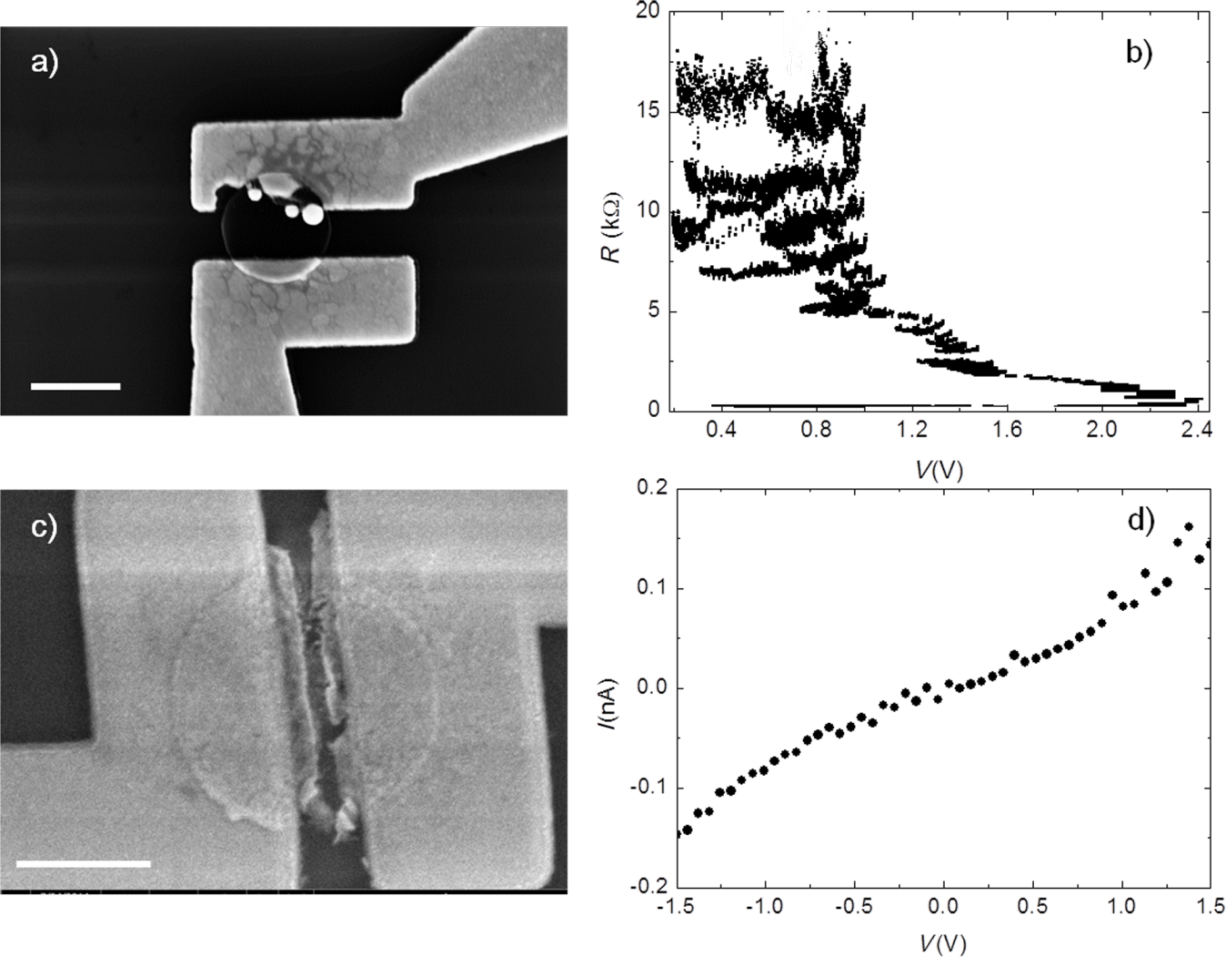
a) SEM image of a non-patterned disc after the EB process. During EB the area around the graphene–metal contact gets heavily damaged due to high power dissipation at these spots. b) EB cycle for a patterned TG disc (see text) showing the transition from low-ohmic (≈200 Ω) to high-ohmic (≈20 kΩ) behavior, which indicates the opening of a gap. c) Corresponding SEM image showing intact metal contacts while a breaking is visible in the disc. d) *I*–*V*-characteristic of electroburned TG device. A tunneling current is visible, demonstrating the presence of an open gap in the range of a few nanometers only. The scale bar is always 1 µm.

In a first attempt, simple pairs of source–drain electrodes have been defined on the discs by electron beam lithography. More than 10 discs have been contacted and underwent the EB process in air. In all of these experiments the EB took place at the contact areas as exemplary shown in Figure 5a and it was not possible to reach sufficiently high current densities to initiate any EB process. Instead, more power was dissipated where the resistance was higher due to the additional contact resistances. This turned out to occur most likely close to damaged electrodes. In all of these devices, no current is measured after the EB process. In order to overcome this problem and to obtain a high current density in the interior of the discs, we pre-patterned them before making the electrical contacts, thus defining a constriction inside the discs (see Experimental for details on the lithography procedure). The narrowest spot now gives the largest current density when a bias is applied. Indeed, we find that the EB process in those devices is well-controlled up to resistances of around 16 kΩ before it opens up a gap and the resistance eventually jumps to higher values showing that one can obtain controlled contact dimensions. Figure 5b shows how the resistance evolves over several EB cycles. A cycle is defined as the increase of bias voltage until either a target voltage is reached or the resistance increases by a certain percentage and then starting again from a voltage level that corresponds to a defined fraction of dissipated power in the device. The resulting gap at the desired position close to the disc center rather than at the contacts is shown in Figure 5c. We measured four patterned discs and all of them showed a similar behavior. Measuring the *I*–*V*-characteristics of such an electroburned device can verify the actual presence of a gap. Typically a bias voltage of 1 V results in a current of 80 pA, as exemplified in Figure 5d showing a typical tunneling current through the gap in the disc.

## Discussion

We now compare the different behaviors encountered in the three types of graphene analyzed. Concerning the exfoliated few-layer graphene flakes, we found that performing the EB in vacuum leads to a higher yield of nanometer-gap fabrication. At the same time the EB process happens at higher voltages under vacuum than in air, the current being almost identical under the two conditions. This means that the dissipated electrical power and the local temperature of the devices are similar or even slightly higher in the case of vacuum. Since a higher local temperature is usually considered to be detrimental for a controlled gap formation, we conclude that the environment pressure must be the key parameter to optimize the EB process of few-layer graphene on SiO_2_ and achieve a better control on the gap formation.

Graphene grown on the C-face of SiC exhibits a similar behavior under ambient conditions, while under vacuum the oxygen–carbon reaction does not even initiate. This difference must be related to the different stacking feature (turbostratic vs graphite-like) of the graphene itself and/or to the different characteristics of the graphene/substrate systems (exfoliated graphene/SiO_2_ vs graphene/SiC).

Focusing on these parameters, it is interesting to consider the case of turbostratic microdiscs, which have a morphology similar to that of graphene on SiC but are deposited on the same substrate as the exfoliated graphene. Here, we found that the EB process leads to a breaking of the graphene devices only after the patterning of a constriction. This hardness with respect to exfoliated graphene suggests that the different morphology of the edges also plays a role to initiate the burning. Indeed, the presence of nonsaturated carbon bonds makes the edges the most reactive part of the device. Edges cleaved during the exfoliation (exfoliated graphene), edges created during the oxygen plasma (graphene on SiC and turbostratic discs after the patterning), and edges of the untreated microdiscs [25] do have different configurations and therefore a different propensity to ignition. Moreover, we expect a higher presence of impurities when using exfoliated graphene on SiO_2_ (which is known to be more reactive than SiC). Such impurities may be working as catalysts, initiating the EB process of graphene even under a reduced oxygen atmosphere.

## Conclusion

We presented a systematic study of the electroburning (EB) process in few-layer graphene devices for different graphene types. We focused our attention on exfoliated graphene, graphene epitaxially grown on the C-face of SiC and turbostratic graphene microdiscs comparing the results obtained when working in air and under vacuum. We showed how the process strongly depends on the specific type of graphene and on the environment pressure in the chamber. For mechanically exfoliated graphene, the vacuum process leads to the formation of nanometer-sized gaps with a higher yield and generally smaller sizes. On the other hand, for graphene on the C-face of SiC, the EB process creates a gap in the graphene devices when it is performed in air, while under vacuum conditions it simply leads to the blow-up of the metal contacts. As-deposited turbostratic graphene discs are found to be extremely resilient against the EB process: The opening of a gap in the device is possible only after creating a hot spot in the discs, as demonstrated in our work after the patterning of a constriction.

Our work suggests that the oxygen pressure is a key factor in the EB process but also other factors such as the type of graphene stacking, the morphology of the edges and the specific graphene/substrate system play an important role. Further studies will focus on tuning the partial oxygen pressure during the electroburning to determine the optimal working conditions for the different types of device. We believe that these results will contribute to the realization of reliable graphene based electrodes for molecular electronics and spintronics.

## Experimental

Few-layer graphene flakes were deposited by the standard “scotch tape” mechanical exfoliation method from natural graphite pieces on top of a p-doped silicon wafer coated with 300 nm of oxide. Flakes of suitable thickness (1 to approx. 20 layers) were located with an optical microscope on the basis of their contrast with the substrate. In some cases, the effective number of layers is also checked by micro-Raman spectroscopy, see Supporting Information File 1 for some examples. Metal contacts (10 nm Cr/100 nm Au) on the graphene sheets have been obtained by electron beam lithography (EBL), thermal evaporation and lift-off in acetone.

Turbostratic graphene was obtained on on-axis SiC(000−1) semi-insulating wafer dice. First, the SiC dice were hydrogen-etched in order to obtain atomically flat surfaces [31]. This process was carried out in a resistively heated cold-wall reactor (high-temperature Aixtron BM) at a temperature of 1350 °C, a pressure of 450 mbar, for 10 min. Subsequently, graphene was obtained in the same reactor through thermal decomposition of SiC [30] under an argon atmosphere, heating at 1420 °C for 90 min. Attenuation of the SiC signal in Raman spectroscopy was used to estimate the number of grown layers, which were found to be about ten. Also, combined Raman and atomic force microscopy (AFM) indicated a good homogeneity and quality of the grown graphene. More details are given in the Supporting Information File 1.

To assure good ohmic contacts, the first fabrication step was the thermal deposition of 3 nm Cr/30 nm Au as the initial metal contacts. Successively, graphene was patterned in the desired device geometry (two-probe device, the graphene channel is roughly 1 × 3 μm) by electron beam lithography and oxygen plasma etching (30 s in a Diener Femto plasma system at maximum power). Finally, the remaining metal parts (the bonding pads and the connections from the contacts to the pads) were obtained by the evaporation of 10 nm Cr/100 nm Au and lift-off.

For mechanically exfoliated and epitaxial graphene the electroburning process was performed by applying an increasing voltage ramp (20 mV/s) while continuously measuring the conductance of a junction. The burning of carbon atoms initiated the formation of a gap and increased the resistance. Once the resistance overcome the chosen value of 200,000 Ω, the voltage was immediately reset to zero (<100 µs). The process was performed at room temperature either in air or under vacuum (<10^−4^ mbar). After the electroburning process, *I*–*V* measurements were taken with an AdWin-Pro system (16 bit output and input ) using a FEMTO pre-amplifier.

Turbostratic multilayer graphene discs were grown in large quantities by the pyrolysis of hydrocarbons in a plasma torch process. The graphitic discs were dispersed in 1-methyl-2-pyrrolidinone by using bath sonication followed by centrifugation allowing for the separation of the discs from each other and from other types of microstructure [25]. The material was then dried in the form of powders, which were then deposited on a p-doped silicon wafer coated with 300 nm of oxide by an adhesive tape. After rinsing with acetone and isopropanol, hundreds of discs were left on the surface. Typical Raman spectra of the as-deposited discs are shown in the Supporting Information File 1. The discs were located by an optical microscope and then patterned in the hour-glass geometry by oxygen plasma (2–3 min depending on the discs thickness, for such a long etching time we had to use a 800 nm thick PMMA layer) and finally electrically contacted with thermally evaporated 10 nm Cr/100 nm Au by electron beam lithography and lift-off. After the PMMA development and immediately before the metal evaporation, a short (<10 s) plasma step was performed to assure good ohmic contacts on the discs. Electroburning and *I*–*V* tests were made under ambient conditions by using a Keithley voltage source and femtoamperometer in the two-probe configuration.

## Supporting Information

See Supporting Information for the Raman spectra of the different graphene materials (exfoliated, graphene on the C-face of SiC, turbostratic micro-discs deposited on SiO_2_); the AFM characterization of the graphene grown on SiC; additional details about the fitting procedure of the non linear *I*–*V* curves according to the Simmons model.

File 1 Titel: Material characterization.
