# Supplementary material for: Electroburning of few-layer graphene flakes, epitaxial graphene, and turbostratic graphene discs in air and under vacuum
Source: Beilstein J Nanotechnol. 2015 Mar 11;6:711–9. doi: 10.3762/bjnano.6.72 (PMC4362043; doi:10.3762/bjnano.6.72)
Supplement: File 1 — Titel: Material characterization. [file Beilstein_J_Nanotechnol-06-711-s001.pdf]

Supporting Information

for

Electroburning of few-layer graphene flakes, epitaxial graphene, and turbostratic graphene discs in air and under vacuum

Andrea Candini*[1], Nils Richter[2,3], Domenica Convertino[4], Camilla Coletti[4], Franck Balestro[5,6], Wolfgang Wernsdorfer[5], Mathias Kläui[2,3] and Marco Affronte[1,7]

Address:

[1]Centro S3, Istituto Nanoscienze – CNR, Via Campi 213/a, 41125 Modena, Italy;

[2]Johannes Gutenberg Universität-Mainz, Institut für Physik, Staudinger Weg 7, 55128 Mainz, Deutschland;

[3]Graduate School of Excellence Materials Science in Mainz (MAINZ), Staudinger Weg 9, 55128 Mainz, Deutschland;

[4]Center for Nanotechnology Innovation @ NEST, Istituto Italiano di Tecnologia, Piazza San Silvestro 12, 56127 Pisa, Italy;

[5]Institut Néel, CNRS and Université Joseph Fourier, B.P. 166, 38042 Grenoble Cedex 09, France;

[6]Institut Universitaire de France, 103 boulevard Saint-Michel, 75005 Paris, France;

[7]Dipartimento di scienze Fisiche Informatiche e Matematiche, Università di Modena e Reggio Emilia, Via Campi 213/a, 41125 Modena, Italy;

Email: Andrea Candini - andrea.candini@nano.cnr.it

* Corresponding author

Material characterization

Exfoliated graphene

In Figure S1 we show two examples of the Raman spectra of our multilayer graphene flakes. The absence of the D peak at 1300 cm^{-1} indicates a general good quality of our samples. We firstly identified the suitable flakes (number of layers from 1 to approx. 20) on the basis of their color contrast with the substrate at the optical microscope and for some of them we checked the quality and the effective number of layers with Raman spectroscopy. Figure S1 a) displays a typical example for a thin flake (number of layer < 5) and in b) a typical thicker multilayer (>5) samples is presented. Note that the Raman signal for the sample in b) is similar but not yet identical to the one associated to bulk graphite (see [1] for a reference).

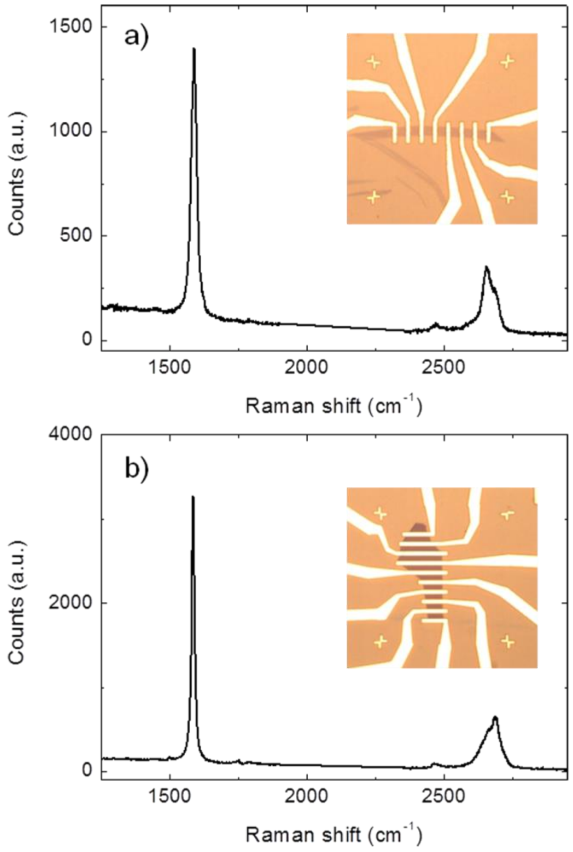

Figure S1: Examples of Raman spectra for the graphene flakes used in this study taken with a laser excitation of 632 nm. The corresponding devices are shown in the insets. a) Thin flake with number of layers < 5. b) Thicker multilayer graphene flake. See [1] for a comparison with the Raman spectra of graphene flakes of various thicknesses.

Epitaxial turbostratic graphene on the C-face of SiC

In Figure S2 we report the AFM and Raman characterization of a typical sample of graphene grown on the C-face of SiC studied in this work. The average roughness in the AFM image is 1.2 nm, which means that the observed steps are mostly single-atom thick. The attenuation of the SiC signal in the Raman spectrum is used to estimate the number of layers, which is found to be around ten [2].

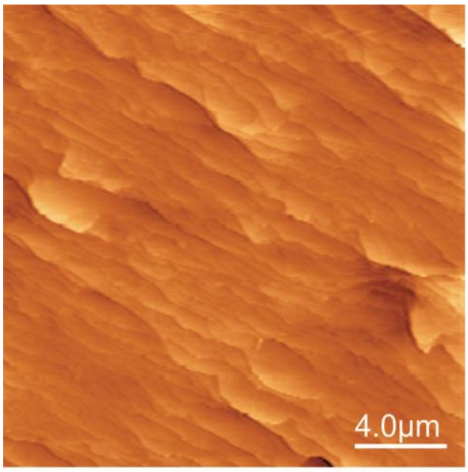

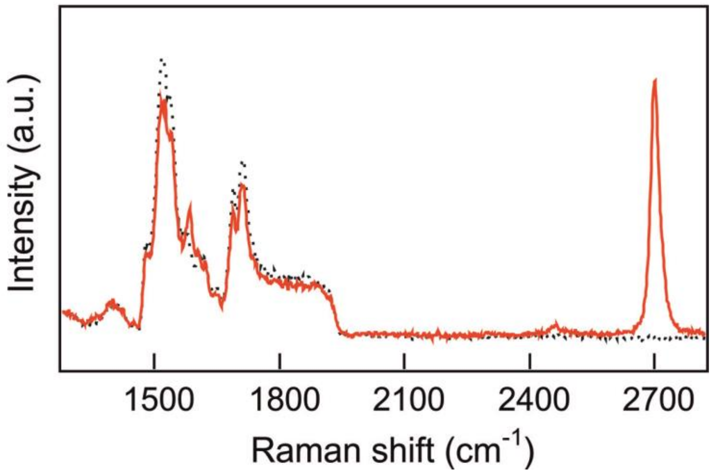

Figure S2: Left: AFM image of a typical graphene area. Right: Raman spectra of the same SiC region before (black dots) and after (red line) the graphene growth. The attenuation of the SiC signal is used to estimate the number of graphene layers. The laser excitation is 514 nm.

Turbostratic graphene discs

In Figure S3 we present a typical Raman spectrum measured on a single turbostratic disc deposited on SiO_2 substrate. The signal is analogous to what previously reported for this material [3].

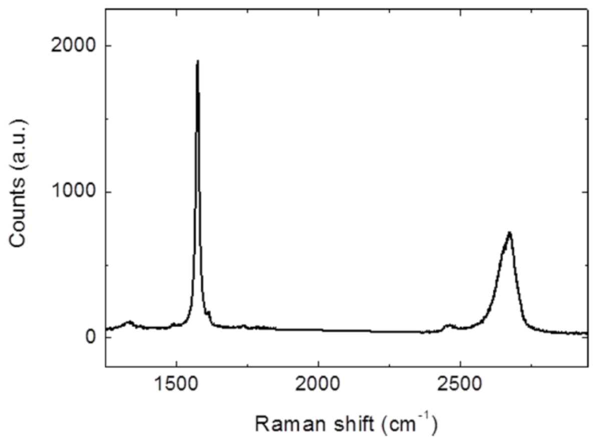

Figure S3: Typical Raman spectrum for a single carbon disc deposited on 300 nm SiO_2 recorded at 632 nm excitation wavelengths.

Details on the Simmons fitting procedure

The nonlinear *I*–*V* curves measured after the electroburning were fitted according to the Simmons model using the following formula for the current *I* [4,6]:

$$I = \frac{Ae}{4\pi^2\hbar d^2}\left[\left(\phi - \frac{eV}{2}\right)e^{\frac{-2d}{\hbar}\sqrt{2m}\sqrt{\phi-\frac{eV}{2}}} - \left(\phi + \frac{eV}{2}\right)e^{\frac{-2d}{\hbar}\sqrt{2m}\sqrt{\phi+\frac{eV}{2}}}\right] \quad \text{(Equation S1)}$$

where *A* is the area of the junction, *e* is the electron charge, $\hbar$ is the reduced Planck's constant, *d* is the gap size, Φ is the height of the tunnel barrier (assumed to be rectuangular), *m* is the electron mass and *V* is the applied potential. This model uses three free parameters for the fitting: the junction area *A*, the barrier height Φ and the gap size *d*. It is known that *A* and Φ are quite sensitive and their values may strongly depend on the initial parameter set, but *d* is more robust and actually more reliable as a fitting parameter.

As an example, in Figure S4 we show the *I*–*V* curve from Figure 1,b of the main text along with three curves calculated from Equation S1 using different sets of parameters, all showing a similar agreement with the experimental data. The first fit (red line) is obtained with *A* = 500 nm^2, Φ = 0.9 eV, *d* = 1.97 nm; the second fit (green line) with *A* = 5 nm^2, Φ = 0.6 eV, *d* = 1.83 nm; the third fit (blue line) with *A* = 1 nm^2 Φ = 0.5 eV, *d* = 1.79 nm. While the values of the A and Φ parameters can change significantly, the value obtained

for d has a much narrower distribution. This is a direct consequence of the fact that the current depends exponentially on the gap size. For all of our measured devices, the optimal values for *d* are between 0.5 nm and 3 nm. These values strongly support our conclusion that the gap size is in the nanometer range for those devices where a tunneling current is measured after the electroburning process.

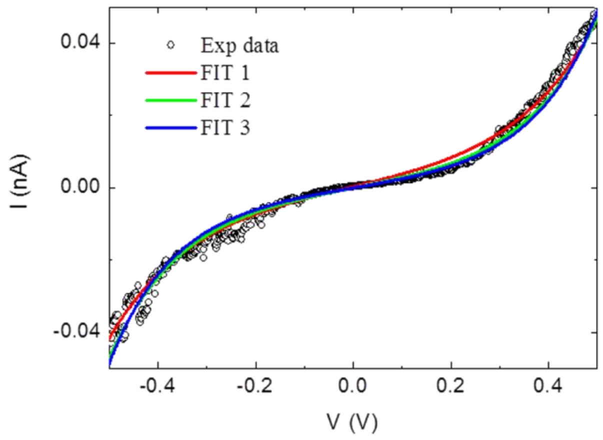

Figure S4: *I*–*V* curves fitted using Equation S1 with three different set of parameters. Fit 1 is obtained with A = 500 nm^2, Φ = 0.9 eV, d = 1.97 nm; fit 2 with A = 5 nm^2, Φ = 0.6 eV, d = 1.83 nm; fit 3 with A = 1 nm^2 Φ = 0.5 eV, d = 1.79 nm.

References

1. Ferrari, A. C.; Meyer, J. C.; Scardaci, V.; Casiraghi, C.; Lazzeri, M.; Mauri, F.; Piscanec, S.; Jiang, D.; Novoselov, K. S.; Roth, S.; Geim, A. K. *Phys. Rev. Lett.* **2006,** *97,* 187401. doi:10.1103/PhysRevLett.97.187401
2. Shivaraman, S.; Chandrashekhar, M. V. S.; Boeckl, J. J.; Spencer, M. G. *J. Electron. Mater.* **2009,** *38,* 725–730. doi:10.1007/s11664-009-0803-6
3. Hernandez, Y.R.; Schweitzer, S.; Kim, J.-S.; Patra, A. K.; Englert, J.; Lieberwirth, I.; Liscio, A.; Palermo, V.; Feng, X.; Hirsch, A.; Kläui, M.; Müllen, K. arXiv:1301.6087, **2013**.
4. Simmons, J. G. *J. Appl. Phys.* **1963,** *34,* 1793. doi:10.1063/1.1702682

5. Prins, F.; Barreiro, A.; Ruitenberg, J. W.; Seldenthuis, J. S.; Aliaga-Alcalde, N.; Vandersypen, L. M. K.; van der Zant, H. S. J. *Nano Lett.* **2011,** *11,* 4607–4611. doi:10.1021/nl202065x
6. Nef, C.; Pósa, L.; Makk, P.; Fu, W.; Halbritter, A.; Schönenberger, C.; Michel, C. *Nanoscale* **2014,** *6,* 7249–7254. doi:10.1039/c4nr01838a
